# Transcription-induced domains form the elementary constraining building blocks of bacterial chromosomes

**DOI:** 10.1038/s41594-023-01178-2

**Published:** 2024-01-04

**Authors:** Amaury Bignaud, Charlotte Cockram, Céline Borde, Justine Groseille, Eric Allemand, Agnès Thierry, Martial Marbouty, Julien Mozziconacci, Olivier Espéli, Romain Koszul

**Affiliations:** 1 Institut Pasteur, CNRS UMR 3525, Université Paris Cité, Unité Régulation Spatiale des Génomes, Paris, France; 2 Collège Doctoral, Sorbonne Université, Paris, France; 3 Center for Interdisciplinary Research in Biology (CIRB), Collège de France, CNRS, INSERM, Université PSL, Paris, France; 4 INSERM–U1163, Unité mécanismes cellulaires et moléculaires des désordres hématologiques et implications thérapeutiques, Institut Imagine, Paris, France; 5 Laboratoire Structure et Instabilité des Génomes, UMR 7196, Muséum National d’Histoire Naturelle, Paris, France

**Keywords:** Transcription, Cellular microbiology, Nuclear organization, Chromosomes

## Abstract

Transcription generates local topological and mechanical constraints on the DNA fiber, leading to the generation of supercoiled chromosome domains in bacteria. However, the global impact of transcription on chromosome organization remains elusive, as the scale of genes and operons in bacteria remains well below the resolution of chromosomal contact maps generated using Hi-C (~5–10 kb). Here we combined sub-kb Hi-C contact maps and chromosome engineering to visualize individual transcriptional units. We show that transcriptional units form discrete three-dimensional transcription-induced domains that impose mechanical and topological constraints on their neighboring sequences at larger scales, modifying their localization and dynamics. These results show that transcriptional domains constitute primary building blocks of bacterial chromosome folding and locally impose structural and dynamic constraints.

## Abstract (web summary)

Here, by sub-kb Hi-C and chromosome engineering, the authors visualize bacterial transcriptional units, showing that they form transcription-induced domains. Transcription-induced domains enforce constraints on nearby sequences, affecting their localization and dynamics.

## Main

Bacterial genomes are organized into the nucleoid, a well-defined membrane-less compartment where DNA, RNA and proteins interact to shape the conformation of chromosome(s)^1–3^. DNA opening, associated with replication and transcription, modulates transiently the supercoiling level of the DNA fiber^4^ by creating twin domains spanning 25 kb in each direction^5^. Topoisomerases, mainly Topo I and DNA gyrase, maintain supercoiling homeostasis, to keep the negatively supercoiled state necessary for DNA compaction and strand opening operations^6^. Radial plectoneme loops are proposed to decorate the bacterial chromosomes, either in association with protein complexes of the structural maintenance of chromosome (SMC) family^7,8^ or with supercoil-induced processes^9,10^. Hi-C contact maps have also revealed higher-order levels of organization in bacterial chromosomes^11–15^, with directionality index (DI) analysis (a statistical parameter that assesses the degree of upstream or downstream contact bias for a genomic region) pointing at ~30 chromosome self-interacting domains (or CIDs) ranging in size from ~30 to 300 kb (ref. ^11^). A careful analysis further unveiled a correlation between highly expressed (and long) genes (HEGs) and CID boundaries, although it was not systematic^12,14^. A short-range correlation was also described between the transcription level and the contact frequencies between pairs of adjacent, 5-kb DNA segments (bins)^13^. Furthermore, inhibition of transcription initiation by rifampicin abrogates domains and decondense nucleoids within minutes, suggesting a direct role for transcription in folding the chromosome^16,17^. On top of supercoiling generation, other transcription-related effects can influence the chromosome conformation. Recent experiments and biophysical models revealed that RNA production reduces the effective solvent quality of the cytoplasm and consequently impacts the local conformation of the DNA fiber^18^. However, with respect to the scale of gene and operon (<10 kb)^9^ of bacterial genomes, these analyses remain relatively coarse. In addition, gene density, concomitant transcription and cell-to-cell variability of hundreds of genes could lead to intermingled patterns, leaving the possibility that fundamental underlying structural features have been overlooked.

In this Article, we combine a high-resolution Hi-C protocol recently adapted for bacteria^19^ with chromosome engineering and cellular imaging to address the link between chromosome architecture and transcription at a higher level. We show that all active transcription units (TUs) form discrete individual, insulated three-dimensional (3D) domains that form the primary building blocks for larger chromosome folding.

## Results

### High-resolution Hi-C reveals transcription-associated contacts

**Fig. 1:**
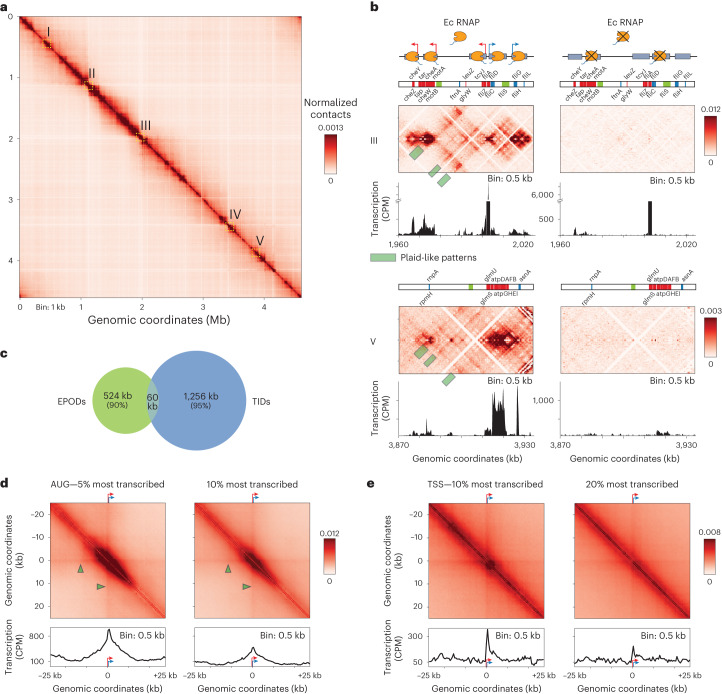
The bacterial chromosome is structured by tens of small transcriptionally active 3D units. **a**, Hi-C normalized contact map of WT *E. coli* cells (bin: 1 kb). The five yellow squares I–V underline representative 64-kb regions magnified in either **b** or in Extended Data Fig. 1. **b**, Magnifications of regions III and V in absence (left) and presence (right) of rifampicin. Ec RNAP: *E. coli* RNA Pol II. For each window and condition: Top: a schematic representation of the region’s genetic content, with the names of genes within the 10% most transcribed indicated in blue and red for forwards and reverse orientation, respectively, and silent EPODs regions in green^21^. Middle: normalized contact map (bin: 0.5 kb). Bottom: RNA-seq profile in CPM. Plaid-like pattern positions are pointed with greenish rectangles on the maps. **c**, Venn diagram of EPODs labeled regions^21^ and of regions labeled as bundle domain. The metric used corresponds to the total size of the corresponding regions, in kb. **d**, Top: pileup of 50 kb contact map windows (bin: 0.5 kb) centered on the start codons (AUG) of the 5% (left) and 10% (right) most transcribed genes of the genome. Bottom: corresponding pileup of transcription (RNA-seq) tracks. Green arrowheads indicate a faint stripe signal extending from the TSS. **e**, Pileup of 50-kb contact map windows centered on the TSS of the to 10% and 20% most transcribed TUs (that is operons). Bottom: corresponding pileup of transcription (RNA-seq) tracks.

**Extended Data Fig. 1:**
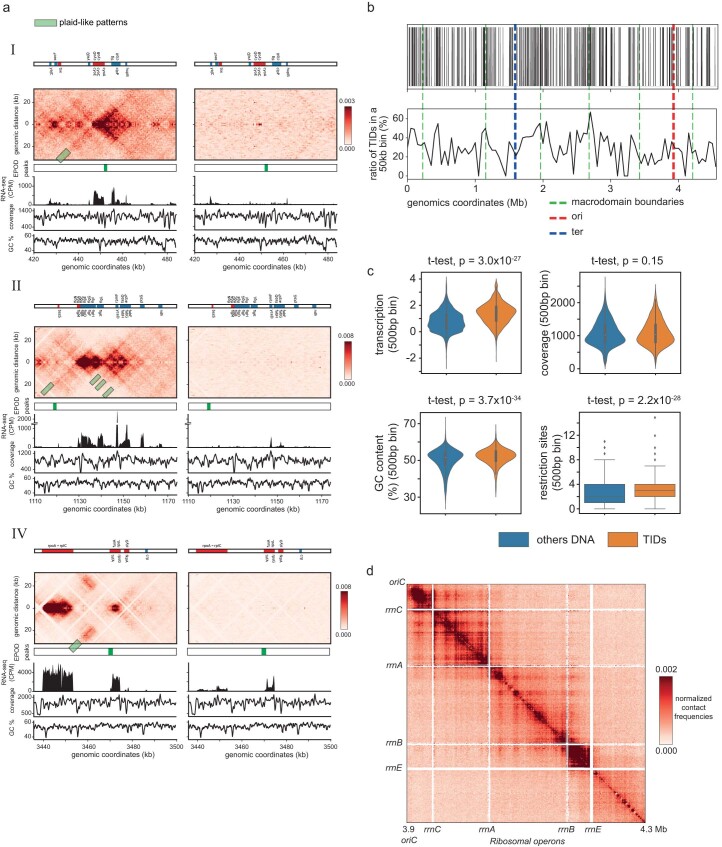
Transcription impact on WT bacterial chromosome folding. **a**, Magnifications of regions I, II and IV. The names of the genes within the 10% most transcribed are indicated (blue and red correspond to forwards and reverse genes, respectively). Left panels: normalized contact map (bin: 0.5 kb) over the corresponding EPODs^12^ peaks and RNA-seq profile (in count per million or CPM), Hi-C coverage, GC content (%), in absence of rifampicin. Right panel: same region and analysis but in presence of rifampicin. **b**, Distributions of the bundle domains across the whole genome (x axis). Top: each strip represents a 500 bp bin called within a bundle domains (that is TID; Methods). Bottom: same data as above but binned into 50 kb bins. The positions of the macrodomains as defined in Lioy et al.^8^ are indicated by green dotted lines. Ori and ter are indicated by red and blue lines, respectively. **c**, Distributions of transcription (CPM, in log 10), coverage, GC content and numbers of restrictions sites in pairs of bins with either low (blue) and high (that is in TIDs; orange) contact frequency at short range (Methods). Boxplots represent the first quantile, the median and the third quantile and the bar is between the first and ninth decile. The p-values are from independent one-sided t-tests (Others DNA: n = 6772, TIDS: n = 2512). **d**, Magnification of the WT *E. coli* contact map binned at 1kb on the rDNA loci. rDNA positions are indicated with their names. As rDNA operons are repeated sequences, reads cannot be mapped unambiguously, resulting in no signal is these loci (white lines in the contact map).

High-resolution (0.5 or 1 kb) Hi-C contact maps of exponentially growing *Escherichia coli* cells reveals strong heterogeneity in the short-range contact signal (Fig. 1a and Methods), with ~200 short regions exhibiting strong and dense short-range signal, subsequently referred to as bundled domains (for calling of these regions, see Methods). These patterns, which cover approximately 1,300 kb, are strongly correlated with transcriptional activity and disappear upon addition of rifampicin (Fig. 1b and Extended Data Fig. 1a). They range in size from 1 to 20 kb and are distributed over the entire genome map (Extended Data Fig. 1b). The potential to make protein–DNA crosslinks will influence local Hi-C contacts^20^. Therefore, the local protein concentration on the DNA (protein occupancy) may contribute to the local Hi-C bundled signal. We took advantage of recent high-resolution maps of protein occupancy on the *E. coli* genome^19^ to test whether silent regions nevertheless strongly enriched in proteins (EPODs) would appear as bundled domains in Hi-C maps. As shown on Fig. 1c, only ~10% of EPODs regions appear to be involved in a bundled domain, suggesting that protein occupancy per se is not sufficient to promote their formation. Overall, the positioning of the bundled domains are not correlated with Hi-C coverage, nor protein occupancy as quantified in ref. ^21^, suggesting that they do not correspond to DNA regions that are more visible or captured by the Hi-C protocol (Extended Data Fig. 1b,c and Methods)^22^.

In addition, a plaid-like pattern was often observed, corresponding to enrichment in contacts between successive transcribed DNA regions, alternating with nontranscribed regions with which they make fewer contacts (Fig. 1b and Extended Data Fig. 1a). The pattern was even more pronounced in the origin region that contains four ribosomal operons (Extended Data Fig. 1d). These contacts involved both ribosomal operons and highly expressed protein-coding genes. These observations suggest that neighboring transcribed regions tend to contact each other locally, either because they may relocate to the nucleoid external periphery, as suggested by super-resolution imaging^17,23,24^, or through an unknown transcription-dependent clustering mechanism.

To further quantify the correlation between local Hi-C contacts and gene expression, a pileup analysis of the averaged contacts centered on the start codon of the 5% and 10% most transcribed genes was performed (Fig. 1d). A bundled signal centered on the start codon appeared, strongly correlated with the corresponding averaged transcription signal (Pearson correlation 0.81). Because bacteria genes are often organized into operons and cotranscribed, we then plotted the pileup contact windows centered on the start codon of the first gene of the most transcribed operons (transcription start site, TSS) (Fig. 1e and Methods). The pileup displays an enrichment in local Hi-C contacts that increases abruptly precisely at TSS positions, and extends over the area spanned by the transcription track, further reinforcing the notion that short-range (0–5 kb) Hi-C contacts are correlated with transcription levels (Pearson correlation 0.62). Faint stripes crossing the map highlight slight enrichment of contacts between the TU and upstream and downstream regions is also observed, a signal that corroborates the plaid-like pattern observed on the sub-kb contact map (Fig. 1d,e, pointed at by green triangles).

Taken together, these observations suggest that the primary blocks organizing the *E. coli* chromosome consist of a succession of bundled domains, that make short-range contacts in Hi-C maps, and which we term transcription-induced domains (TIDs). TIDs are separated by nontranscribed regions depleted in local Hi-C contacts but can interact together as long as the genomic distance between them remains relatively small (with the present experimental approach: <25 kb).

### TIDs explain CIDs detection in low-resolution maps

**Extended Data Fig. 2:**
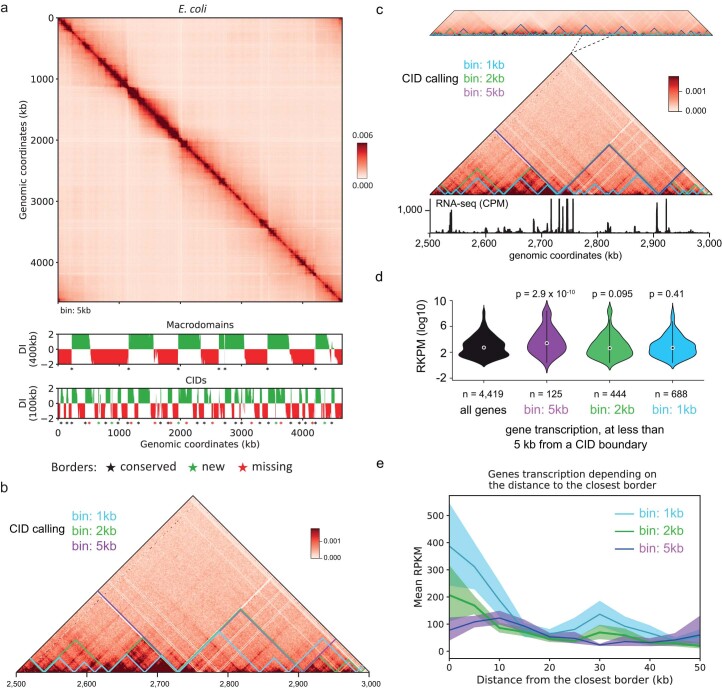
Relationships between Transcription Induced Domains and CIDs. **a**, *E coli* contact map binned at 5 kb at the top. Below the corresponding detected macrodomains and CIDs based on directional index method^9^. Stars show the significant borders detected in both Lioy et al. data^8^ and the present one (black), only in Lioy et al. data (red) and only with our data (green). **b**, Domains detected based on DI analysis only at different resolutions; 1 kb (cyan), 2 kb (green) and 5 kb (blue). **c**, Bundle domains called using insulation score detection at 1 kb (cyan), or DI analysis on contact maps binned either 2 kb (green) and 5 kb (blue). Top, visualization of domains over the entire genome. Middle, magnification of a 500 kb region. Below, Corresponding RNA-seq track in CPM. **d**, Violin plot distributions of transcript levels for all genes in the genome (black), and for all genes in 10 kb windows centered on the domain boundaries called on the 5 kb (blue), 2 kb (green) and 1 kb (cyan) binned maps. The bars represent the first and ninth deciles, and the dots is the mean of each distribution. The p-values of non-parametric one-sided Mann-Withneyu test of whether the later distributions follow a genomewide distribution are indicated. **e**, Gene transcription in RPKM depending on the distance from the closest borders detected at different resolutions. The errors bars are defined as the 95% confidence interval of 1,000 bootstraps.

We next compared the positions of TIDs with the boundaries of CIDs previously identified along the *E. coli* genome^13^. First, we called CIDs in the 5-kb contact map using DI analysis, revealing 27 domains (Methods). Twenty-two of these domains’ boundaries overlapped those previously identified using the same approach^13^, while the others lie at the edge of the detection threshold (Extended Data Fig. 2a–b and Extended Data Table 1). As shown before, these boundaries are enriched with HEGs (Extended Data Fig. 2c–e). The same DI analysis performed over a 2-kb binned contact map yielded 30 new boundaries (green signal, Extended Data Fig. 2b and Extended Data Table 1). Finally, DI analysis proved too noisy when applied on a 1-kb contact map (Extended Data Fig. 2b and Extended Data Table 1). To call CID-like signals in 1-kb contact maps, we adapted HiC-DB, another insulation score approach^25^ (Methods). We detected 135 boundaries, delineating 135 CID-like regions ranging in size from 5 to 125 kb (magenta signal, Extended Data Fig. 2c and Extended Data Table 1). Among those, 22 overlap with the boundaries called with the DI analysis of the 5 kb binned contact map and enriched HEG annotations (blue signal, Extended Data Fig. 2). The remaining 113 positions correspond to less expressed genes (Extended Data Fig. 2d,e). Altogether, these results suggest that the chromosome, rather than being structured into large self-interacting regions, is organized by a succession of short, transcription-induced, compact domains alternating with unstructured regions. This structuring is reminiscent of those observed in budding yeast using the microC technique^26^.

### A single TU is sufficient to imprint a Hi-C domain

**Fig. 2:**
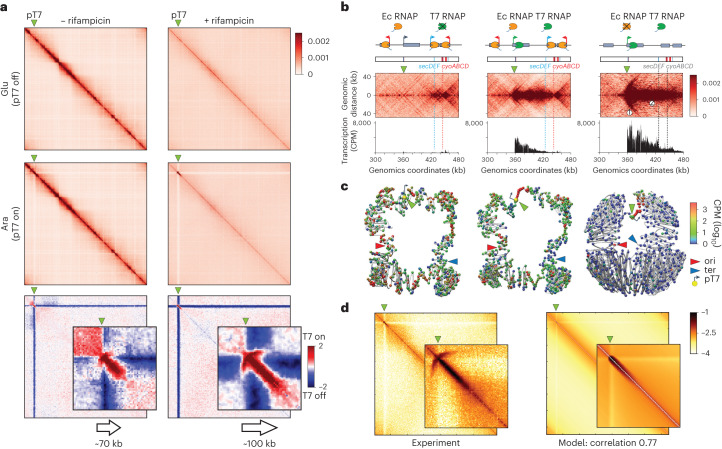
Contact profile of single, active transcription unit within an entire genome. **a**, Hi-C contact maps (bin: 1 kb) of *E. coli* chromosome carrying a single T7 promoter (green triangle), in absence (left) or presence (right) of rifampicin. Ec RNAP: *E. coli* RNA Pol II. Top: cells grown in glucose media, when the T7 RNA Pol is not expressed. Middle: cells grown in presence of arabinose, with expression of the T7 RNA Pol. Bottom: log_2_ ratio contact maps with and without induction of the T7 promoter. Magnification of the T7 promoter region, represented using Serpentine flexible binning (Methods)^48^. **b**, Magnification of the T7 promoter in the normalized contact maps, with and without induction and in presence and absence of rifampicin. From left to right: T7 promoter off, no rif; T7 on, no rif; T7 on, + rif. For each window and condition, a schematic representation of the region’s genetic content is presented on the top, with the operons within the 10% most transcribed indicated in blue and red for forwards and reverse orientation, respectively (that is, *secDEF* and *cyoABCD*). The corresponding RNA-seq tracks (CPM) are plotted under the maps. In presence of T7 RNA Pol and rifampicin, the numbered labels on the map highlight the features discussed in the text: (1) arched stripe pattern; (2) bundle region. **c**, Average genome structures using Shrek of the corresponding 2D contact maps of the *E. coli* bacterial chromosome in the different conditions. The green, red and blue arrows represent the pT7, ori and ter positions, respectively. The 3D representations are not the physical structure of the genome, but the average structure of the population of cells that we observed. **d**, Modeling of the Hi-C contact maps using the RNA Pol distribution on the genome and using the second model (Methods).

**Extended Data Fig. 3:**
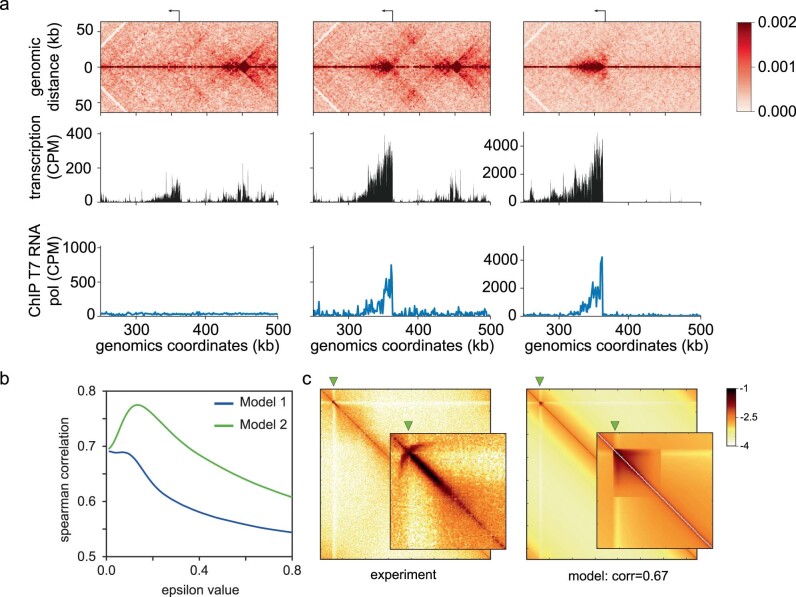
Activation of a single transcription unit within the E. coli chromosome. **a**, Magnifications of the Hi-C contact maps (bin: 1kb) of *E. coli* chromosome carrying a single T7 promoter facing toward the ori, with below the corresponding RNAseq and the signal from ChIP of the T7 RNA polymerase. From left to right: the T7 promoter off, the T7 promoter on and the T7 promoter on with rifampicin. **b**, Correlation between the maps recovered from each of the two models and the experimental map, depending on the epsilon values (Methods). **c**, Best correlation map of Model I (right), aside the experimental map (left).

To further understand the nature of the transcription-dependent, short-range contacts increase observed in the high-resolution contact maps, we designed an artificial inducible system. A T7 promoter was inserted at the *lacZ* locus, facing towards the ter. The T7 RNA polymerase (Pol) is specific to its own promoters and was put under the control of the inducible arabinose promoter (Fig. 2). Upon arabinose addition, a bundled domain appears on the Hi-C map, originating at the pT7 position and propagating towards the ter over ~70 kb when it abruptly stops at the level of the bundled generated by the highly expressed *cyoABCD* operon (Fig. 2a,b). In addition, the Hi-C signal at pT7 is shaped roughly like an arrowhead, while an enrichment in local contacts is also observed upstream the activated promoter. Chromatin immunoprecipitation of the T7 RNA Pol showed a strong enrichment at the pT7 (Extended Data Fig. 3a), whereas RNA sequencing (RNA-seq) analysis further confirmed the strong induction of this artificial TU (Fig. 2b). Since the T7 RNA Pol is insensitive to the bacterial RNA Pol inhibitor rifampicin, we reasoned that treating the cells with the drug should highlight a single transcriptional unit induced by the T7 promoter as genome-wide transcription is turned off^27^. In presence of rifampicin, the chromosome indeed displayed a single TU starting at the pT7 promoter, as determined by RNA-seq and T7 RNA pol chromatin immunoprecipitation followed by sequencing (ChIP–seq; Fig. 2a,b). The absence of neighboring transcription leads to a longer T7 transcription track covering ~110–120 kb, as compared to 70 kb in absence of rifampicin (Fig. 2a,b). This observation further supports the hypothesis that the endogenous *E. coli* RNA Pol of the closest native transcription peak of the *cyoABCD* operon was indeed responsible for blocking T7-induced transcription.

The corresponding normalized contact map displays a clear, discrete bundled domain overlapping the active T7 TU, further magnified when plotting the ratio between the maps of cells treated with rifampicin but with or without T7 induction (Fig. 2c, bottom). Magnification of the induced T7 region from the normalized wild type (WT) with rifampicin map reveals two types of contact pattern at the induced promoter: an ‘arched stripe’, which supplants the rough arrowhead observed earlier and extends from the TSS (Fig. 2b, label (1)) over ~25 kb, and the thick bundled signal that extends across the transcription and T7 RNA Pol deposition tracks (Fig. 2b, label (2); Extended Data Fig. 3a). Consequently, this system allows to magnify a bundled signal emanating from a single TU and decreasing smoothly along a ~110-kb track.

**Extended Data Fig. 4:**
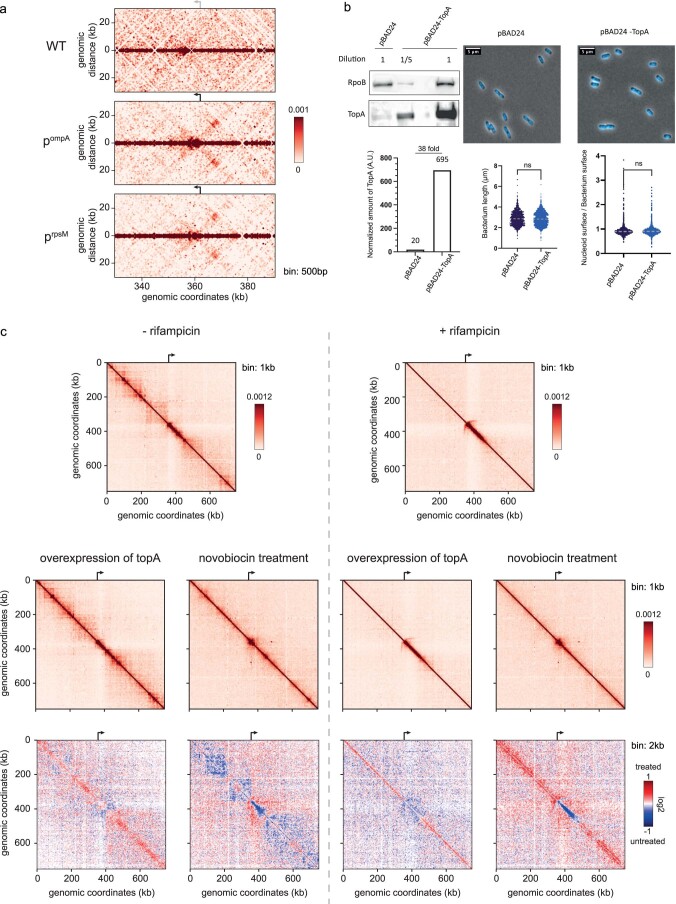
Topoisomerase impact on the transcription unit folding. **a**, Hi-C contact map magnifications (bin: 500 bp) of an *E. coli* strain carrying endogenous promoters facing the origin of replication. From top to bottom: without any additional promoter; with two p^ompA^ promoters; with two p^rpsM^ promoters. **b**, Analysis of TopA overexpression. Left panel, measurement of TopA amount by western blot in RSGB834 pBAD24 and RSGB834 pBAD24-TopA with an anti TopA antibody (gift from Dr. Yuk-Ching Tse-Dinh). Quantification of the western-blot showed a 38 fold overexpression of TopA after 2h of arabinose induction. This experiment was representative of 3 replicates. Right panel, microscopy imaging of the arabinose treated cell. The cells were fixed 2h after arabinose induction and stained with DAPI. Bacteria length and DAPI amount per cell surface was measured with a custom macro of the Omnipose software. The significance of the two-tailed Mann-Whithney test between average of both conditions is indicated by *ns* (not significant) or stars (*: <0.032; **: <0.0021; ***: <0.0002; ****: <0.0001). **c**, Hi-C contact map magnifications of the T7 system while interfering with the topoisomerases activity; contact map binned at 1kb. top; wt system with 2h arabinose treatment. Middle left; overexpression of the topA, right; gyrase inhibition using a 10 min novobiocin treatment. Bottom; log2 ratio of the interfered over the wild type; 2 kb binned. On the left same with 10 min rifampicin treatment. Source data

**Extended Data Fig. 5:**
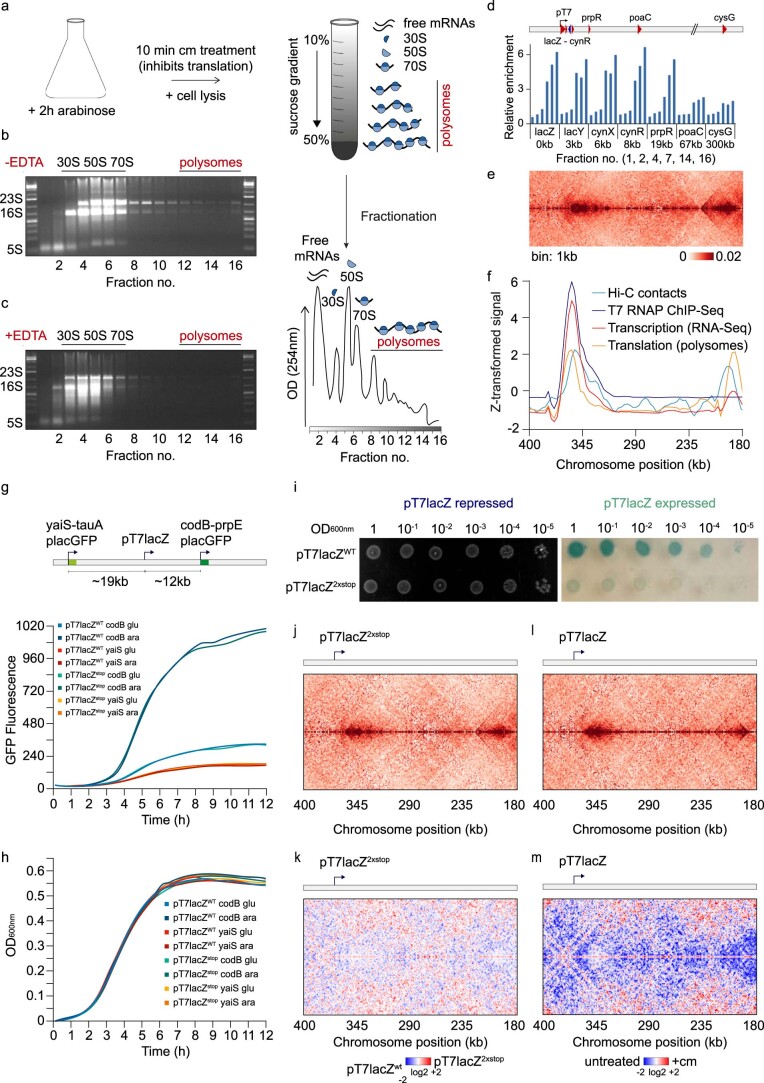
Translation impact on bacterial chromosome folding. **a**, Schematic view of the polysome extraction experiment. **b, c**, Gel migration of the different fractions for polysome extraction without EDTA (b), and with EDTA (c) (ladder: GeneRuler 1 kb Plus DNA Ladder). **d**, Relative enrichment along the chromosome as a function of polysome extraction fraction number. **e**, Magnification of the Hi-C contact map of the *E. coli* carrying T7 promoter facing the origin (oriented from left to right). **f**, Corresponding z-transformed signals of the short range Hi-C signal, T7 RNA polymerase ChIP-seq, transcription and translation. **g**, **h**, Gene expression upstream (*yaiS*) and downstream (*codB*) of the T7 promoter lacZ system with or without STOP codons based on GFP fluorescence (g) and growth of the corresponding strains (h). **i**, Bacterial colony dilution with pT7lacZ repressed on the left and expressed on the right. **j**, Contact map of the bacteria carrying a T7 promoter lacZ system with two stop codons into the lacZ gene. k, Log2 ratio between the contact map with the lacZ2xSTOP system over the contact map with the WT lacZ. **l**, Contact map of the bacteria carrying a T7 promoter lacZ system treated with chloramphenicol. **m**, Log2 ratio between the contact map treated over the untreated.

Both signals were observed upon inversion of the gene (Extended Data Fig. 3a). The bundled pattern, but not the arched stripe, is strongly reminiscent of that observed from the pileup plots of highly expressed TSS of the native genome (Fig. 1d). Using less potent endogenous promoters (*PompA* and *PrpsM*) introduced at the *lacZ* locus, we observed a few kilobases bundles and no obvious arched stripe (Extended Data Fig. 4a). Finally, the transcribed region is covered with polysomes, and thus most likely translated (Extended Data Fig. 5). However, the contact signal is unchanged when (1) two stop codons are introduced downstream the T7 promoter (*pT7lacZ*^*2Xstop*^), preventing the synthesis of the first genes of the T7 TU *lacZYA*, and (2) translation elongation is inhibited by chloramphenicol, a drug that inhibits ribosome translocation and stabilizes messenger RNAs^28,29^ (Extended Data Fig. 5). The former result suggests that the signal could be independent of translation although we cannot exclude a role of ribosomes in TIDs maintenance.

### Modeling TIDs

The data contained in the 2D contact maps can also be visualized in 3D using the shortest-path reconstruction algorithm ShRec3D (Methods). These structures are an alternative, lower-dimension representation of the 2D maps in the 3D space and are based on any physical model. They nevertheless illustrate how the highly transcribed T7 TU contact map forms a discrete structure within the chromosome that appears to insulate flanking regions (Fig. 2d)^30^.

To gain further quantitative insight into the link between transcription and increased short range contacts, we developed two probabilistic modeling approaches to emulate the observed contact map under two different assumptions. In the first hypothesis, the increase in short-range contacts is due to the existence of preferential contacts between T7 RNA Pols that cover the TU. In the second model, we added an insulation effect of the polymerases such that the contact probability between two polymerases decreases if another polymerase is present between them. The models take as inputs the experimentally measured decay in contact frequency with increasing genomic distance and the ChIP deposition profile of T7 RNA Pol. The only fitting parameter, the maximum T7 RNA Pol occupancy along the TU (between 0% and 100%), was set to get the highest correlation between the experimental Hi-C and the model contact maps. The best result (correlation of 0.77) was obtained for the second model and a maximum occupancy of 15% (Extended Data Fig. 3c; compared to a maximum correlation of 0.67 for model 1 in Extended Data Fig. 3c). The extended bundled pattern correlates nicely with the experimental T7 RNA Pol occupancy, suggesting that crosslinking of trains of consecutive RNA Pol along the transcribed track could account for the contact pattern observed (Fig. 2e). These results therefore suggest that the bundled motifs corresponding to TIDs in Hi-C contact maps correspond to trains of RNA Pols that each have a cumulative local insulating effect.

### Interactions between adjacent T7 RNA Pol-induced domains

**Fig. 3:**
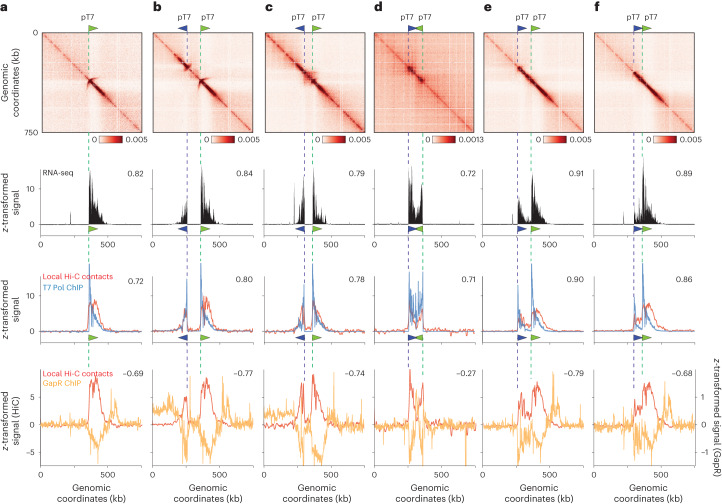
Supercoiling and contacts resulting from combination of pairs of transcription units. **a**–**f**, Genomic characterization of chromosomal regions carrying pairs of pT7 promoters in different orientations. The orientations are as follows: a single promoter (**a**), two divergent promoters at 100 kb (**b**), 60 kb (**c**), two convergent promoters at 100 kb (**d**), two unidirectional (collinear) promoters at 100 kb (**e**) and 60 kb (**f**). From top to the bottom: Hi-C contact map (first row; bin: 1 kb), RNA-seq track (second row; in CPM), T7 RNA Pol ChIP–seq track (third row, blue curve) and short-range Hi-C contacts (third row, red curve), and GapR ChIP–seq revealing positive supercoiling (fourth row, yellow curve) and short-range Hi-C contacts (fourth row, red curve). Values on the top right corner of each panel are the Spearman correlation coefficients of the track with the short-range Hi-C contacts. All tracks are *z*-transformed.

We next combined pairs of transcribed T7 units (*pT7lacZ* and pT7mCherry) to further characterize the potential structural interplay between two neighboring genes. The second pT7 was introduced at either 60 or 100 kb upstream of *pT7lacZ*, either in collinear, convergent or divergent orientation (Fig. 3, first row). Exponentially growing cells were induced for T7 RNA Pol using arabinose, treated with rifampicin and processed with Hi-C and RNA-seq. In all cases, we observed an excellent correlation between the short-range contacts, transcription tracts and T7 RNA Pol as quantified using ChIP–seq (Spearman correlation between 0.62 and 0.91) (Fig. 3, first to third rows). First, and in contrast to the native TUs for which interactions between neighboring TUs are observed (Fig. 1b), no interactions between the pairs of T7 TU were observed (Fig. 3b–f). Second, the arched stripe pattern appears affected by the orientation of the promoters with respect to each other. Upon induction, the two promoters positioned in divergent orientations and separated by 100 kb displayed similar contact patterns, that is, an arched stripe and the bundle signal (Fig. 3b, first and third rows). The arched stripe pattern nevertheless vanished when the distance separating the divergent promoters was shortened (60 kb) (Fig. 3c, first row). Concomitantly, a self-interacting domain of enriched contact emerged in-between the two genes, which strengthens when the distance between the divergent promoters decreases (Fig. 3b,c). These upstream contacts are consistent with the observation made using a single promoter (Fig. 2b). In contrast, the two genes in convergent orientation resulted in the two transcription tracks abruptly ending at mid-distance, resulting in a sharp boundary right in-between the two promoters (Fig. 3d, first to third rows). When positioned in colinear orientation, the well-defined and visible arched stripe of the *pT7lacZ* promoter is strongly reduced, if not entirely suppressed, by the incoming transcription tract of the upstream pT7mCherry (Fig. 3e,f, first to third rows). Transcription induces positive and negative supercoils in front of and behind the RNA Pol^4,5^. These supercoils may indirectly influence the arched stripe by decreasing initiation or elongation by the T7 RNA Pol^31–33^. Because we did not observe dramatic changes of T7 expression besides the abrupt termination of convergent tracks that probably reflect the documented effect(s) of supercoiling on elongation^33^, we favor the hypothesis that an adjacent TU will directly affect the arched stripe because it consists of negative supercoils.

### The arched stripe of the T7 RNA Pol-induced domains

To further explore the nature of the arched stripe, we tested the effects of *topA* overexpression, which encodes for TopoI that actively relaxes negative supercoils into DNA^34^. TopoI overexpression modestly affected the bundle but resulted in 50% reduction of the intensity of the arched stripe signal, suggesting that the latter may result from an accumulation of negative supercoiling upstream of the promoter that overcomes the capacity of the topoisomerase to remove them. By contrast, the inhibition of gyrase, which removes positive supercoil ahead of the RNA Pol, with novobiocin, shortens the transcription bundle signal while concomitantly strongly reducing the arched stripe (Extended Data Fig. 4c). A possibility is that in absence of gyrase the accumulation of positive supercoiling downstream of the track triggers earlier termination of the polymerase, and thus diminishes negative supercoiling upstream of the promoter. To measure the supercoiled nature of the DNA template at the level of the T7 TU(s), we quantified using ChIP–seq the deposition of GapR, a protein of *Caulobacter crescentus* recently introduced as a marker of positive supercoiling along bacterial and yeast chromosomes^35^. In WT *E. coli*, GapR is enriched downstream endogenous active genes^35^. Enrichment of GapR was observed at the 3′ end of the single T7 TU (Fig. 3a, fourth row). This enrichment corresponds to positive supercoils that diffuse over a 50–100 kb region after the T7 TU. No enrichment was observed in-between genes in divergent orientations, but the GapR signal was enriched downstream these transcription tracks (Fig. 3b,c, fourth row), and also in-between genes positioned in convergent orientations (Fig. 3d, fourth row). In colinear orientation, no enrichment was seen after the first gene, in agreement with the suppression of the positive supercoils by the neighboring negative one (Fig. 3e,f, fourth row). However, a strong enrichment was observed after the second gene. Altogether, these results strongly suggest that the arched stripe pattern is the Hi-C signature of a negative supercoiled structure positioned in the upstream 5′ region of the TU. These observations agree with simulated and experimental data pointing at a preferred positioning of RNA Pol at the apical positions of supercoiling loops^36,37^. A fine observation of the signal suggests that, indeed, the T7 promoter is positioned in the middle of the arched stripe. On endogenous genes, this pattern would be either two small to be visualized at the present resolution, erased by neighboring supercoiling (similarly to the collinear T7 units), or most likely both (see Discussion).

### TIDs impose mechanical constraints on adjacent regions

**Fig. 4:**
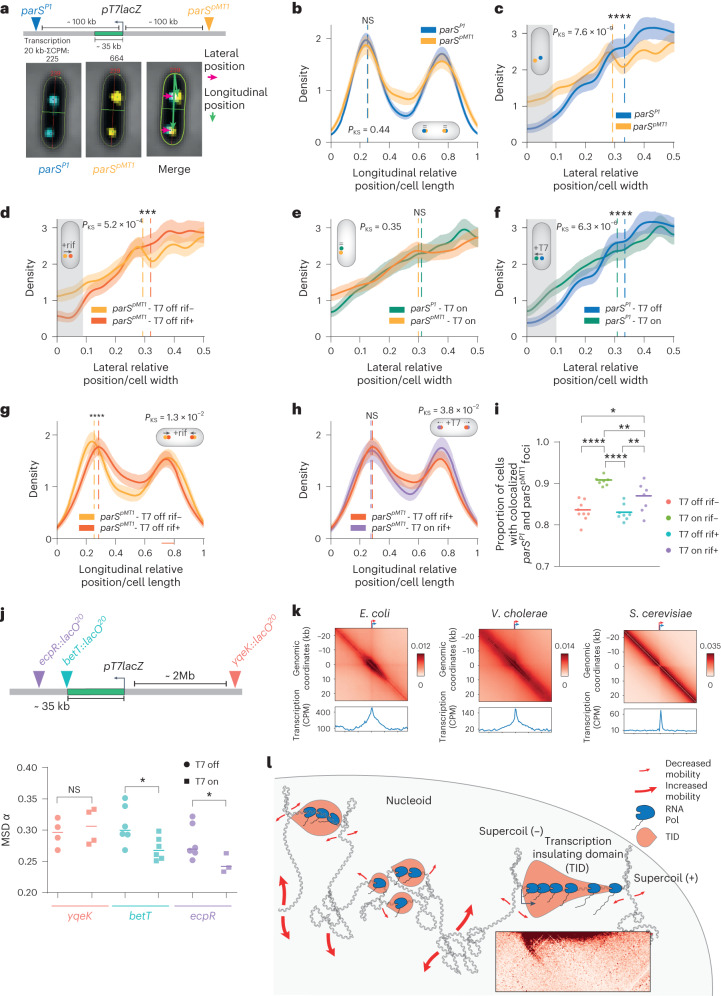
Dynamic influence of the T7 transcription unit. **a**, Positions of the *parS* tags inserted in a TID enriched region (*parS*^*pMT1*^ near *yajQ* gene) and a poorly expressed region (*parS*^*P1*^ near *crl* gene). The T7 promoter was inserted at the lacZ promoter position in-between the two *parS* tags. The arrows on the right indicate how we measure the lateral (pink) and longitudinal (green) positions in **b**–**h** and in Extended Data Fig. 6d–f. In **b**–**h**, the positions of one or two *parS* tags in one or two conditions are compared. The *x* axis represents either the relative longitudinal or lateral relative position. 0 (and 1) corresponds to the cell periphery, whereas 0.5 corresponds to the middle of the cell. For each panel, a cell cartoon illustrates the position that the two monitored loci tend to occupy. Statistical differences between the distributions are analyzed with a two-sample Kolmogorov–Smirnov test. On each plot, the dotted lines indicate the median of the tags positions, and the significance of the one-sided *t*-test between average position of both conditions is indicated by NS (not significant) or stars (*P < 5 × 10^−2^; **P < 1 × 10^−3^; ***P < 1 × 10^−4^; ****P < 1 × 10^−5^). The errors bars are defined as the 95% confidence interval of 1,000 bootstraps. Finally, a gray area can highlight the peripheral localization where and if foci redistribution occurs. **b**,**c**, The longitudinal (**b**) and lateral (**c**) foci positions of *parS*^*pMT1*^ and *parS*^*P1*^ (as shown in **a**), in absence of T7 transcription, are plotted (*t*-test *P* value: 0.62, 8.7 × 10^−8^, respectively). The lateral position of the transcribed *parS*^*pMT1*^ tag region is shifted toward the periphery of the nucleoid cnompared to the inactive *parS*^*P1*^ region. **d**, Lateral position of the *parS*^*pMT1*^ tag in absence or presence of rifampicin (*t*-test *P* value: 8.3 × 10^−4^). **e**, Lateral position of the *parS*^*pMT1*^ and *parS*^*P1*^ tags in the presence of T7 transcription (*t*-test *P* value: 0.12). **f**, Lateral position of the *parS*^*P1*^ tag with or without T7 transcription (*t*-test *P* value: 3.5 × 10^−5^). **g**, Longitudinal position of the *parS*^*pMT1*^ tag in absence or presence of rifampicin (*t*-test *P* value: 1.5 × 10^−5^). **h**, Same as **g** but in presence of T7 transcription (*t*-test *P* value: 6.8 × 10^−2^). **i**, Colocalization of pairs of *parS* tags from **a** flanking the T7 unit. The proportion of cells presenting at least one couple of foci closer than 200 nm of less was plotted. Each replicate is an average of 400 cells. Statistical differences are measured by an analysis of variance (ANOVA) Kruskal–Wallis test with Bonferroni correction, *P < 0.033, **P < 0.0021, ***P < 0.0002, ****P < 0.0001. Replicates: *N* = 7–9. **j**, Positions of the three *lacO* arrays inserted in the vicinity of the T7 promoter. LacI–YFP foci dynamics was analyzed for 100 time intervals of 1 s, for each replicate (*N* = 3–6)) the median MSDα measurement for ~1,000 trajectories of the fluorescently labeled loci was computed. Experiments were performed in the absence of rifampicin upon induction of the T7 TU. Statistical differences are measured by an ANOVA Kruskal–Wallis test with Bonferroni correction, *P < 0.033, **P < 0.0021, ***P < 0.0002, ****P < 0.0001. **k**, *E. coli*, *V. cholerae* and *S. cerevisiae* genes pileups. Left: pileup for each species of 50-kb windows contact maps centered on TSS of the 10% most transcribed genes. Bottom: corresponding RNA-seq pileup profiles. **l**, Schematic representation of the proposed nucleoid structuring into a mosaic of small 3D transcriptional units or TIDs. We propose that TIDs tend to cluster together, and/or relocalize to the nucleoid periphery, resulting in enriched contacts between adjacent units separated by ~20–40 kb. Source data

**Extended Data Fig. 6:**
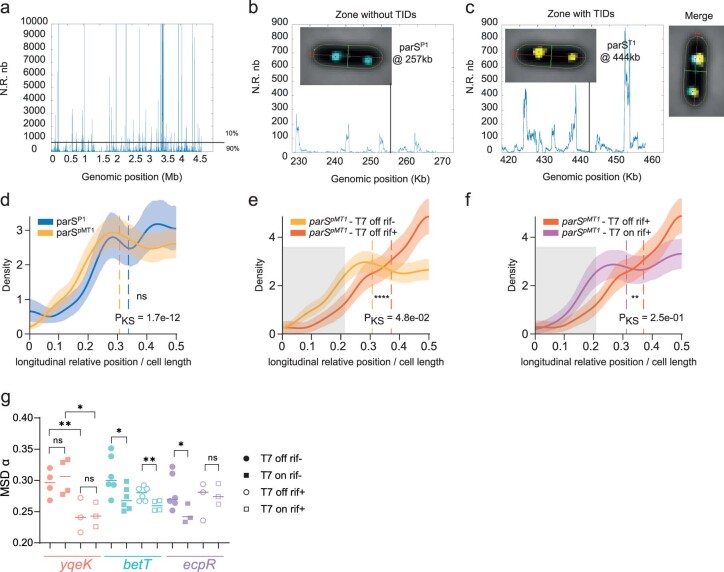
Dynamic influence of the T7 transcription unit. **a**, RNAseq signal over the whole genome. Values are the normalized number of reads at a given position. **b**, Magnification of the RNAseq signal over the *parS*^*P1*^ locus. **c**, Magnification of the RNAseq signal over the *parS*^*pMT1*^ locus. **d-f**, The dotted lines on the plot indicate the median of the loci positions, and the significance of the one-sided t-test between average position of both conditions is indicated by *ns* (not significant) or stars (*: <5.10^−2^; **: <1.10^−3^; ***: <1.10^−4^; ****: <1.10^−5^). The errors bars are defined as the 95% confidence interval of 1,000 bootstraps. The gray area highlights the shift of distributions across conditions. **d**, Longitudinal position of the tags with one focus (t-test p-value: 0.12). **e**, Longitudinal position of the *parS*^*pMT1*^ locus with one focus with or without rifampicin treatment (t-test p-value: 9.8 × 10^−5^). **f**, Longitudinal position of the *parS*^*pMT1*^ locus with one focus with rifampicin treatment is rescued upon T7 activation (t-test p-value: 1.4 × 10^−3^). **g**, Positions of the three *lacO* arrays inserted in the vicinity of the T7 promoter. LacI-YFP foci dynamics was analyzed for 100 time intervals of 1 sec, for each replicate (N = 3-6) the median MSDα measurement for ~1,000 trajectories of the fluorescently labeled loci was computed. Experiment were performed in the presence of rifampicin upon induction of the T7 TU. Statistical differences are measured by an Anova Kruskal-Wallis test with Bonferroni correction, * <0.033, ** < 0.0021, **** <0.0002, ****<0.0001.

To assess the importance of TIDs in living cells, we used fluorescence imaging to monitor two chromosomal regions flanking the T7 promoter. We positioned two markers, separated by 230 kb, one in a ‘silent’ region (*parS*^*P1*^; about 200 normalized RNA-seq reads in the 20-kb flanking region) and the other in a moderately expressed region (*parS*^*pMT1*^; about 700 normalized RNA-seq reads in the 20-kb flanking region; Fig. 4a and Extended Data Fig. 4a–c). The positioning of these regions relative to cell length was similar (Fig. 4b and Extended Data Fig. 6d). However, the lateral positioning of the expressed *parS*^*pMT1*^ region is closer to the nucleoid periphery than the neighboring silent *parS*^*P1*^ region (Fig. 4c) suggesting that endogenous transcription moderately influences gene localization. Rifampicin-induced inactivation of transcription relocalizes the *parS*^*pMT1*^ towards the center of the nucleoid (Fig. 4d). This observation is in agreement with previous findings showing using super-resolution imaging that clusters of RNA Pol tend to (though not systematically) localize near nucleoid periphery, in rich^17^ and minimal^17,24^ medium. Upon transcription activation of the neighboring T7 TU, both loci (silent *parS*^*P1*^ and expressed *parS*^*pMT1*^) localize at the nucleoid periphery (Fig. 4e,f).

In the presence of rifampicin, the longitudinal (Fig. 4g and Extended Data Fig. 6e) and lateral (Fig. 4d) localization of the expressed and silent regions were affected, moving closer to the center. Activation of T7 expression counteracted the effect of rifampicin by moving foci away from the medial position of the cell (Fig. 4h and Extended Data Fig. 6f).

In addition, we studied the impact of transcription on the organization of flanking chromosomal regions. Under natural conditions, approximately 80% of the cell population exhibited colocalization of the two regions separated by 230 kb. However, upon T7 expression, this frequency increased to 90%, regardless of the presence of rifampicin. This suggests that T7-mediated folding influences the convergence of neighboring regions (Fig. 4i).

To monitor the influence of T7 transcription on the mobility of chromosome loci, we used strains carrying fluorescently labeled *lacO*^*20*^ arrays inserted at two positions downstream of the T7 promoter (Fig. 4j). We compared individual foci dynamics with or without T7 induction, in the absence or presence of rifampicin, by recording their position every second for 120 s (Methods). For each trajectory, we computed the mean-squared displacement (MSD), a technique that describes the mode of displacement of particles followed over time. We plotted the slope (*α*) of the MSD versus time interval. *α* is indicative of the nature of the locus movement. *α* = 1 describes normal diffusion, whereas *α* < 1 is subdiffusive. For the two loci close to the T7 TU (*betT* and *ecpR*), T7 activation correlated with a reduction of the *α* median value, suggesting that T7 transcription constraints the movement of the flanking region (Fig. 4j). In contrast, a focus positioned 2 Mb away at the *yqeK* locus did not show notable changes upon T7 activation (Fig. 4j). Note that rifampicin appears to have heterogeneous impact on DNA mobility according to the reporter region monitored, perhaps because of the combination of indirect perturbations (Extended Data Fig. 6g).

Overall, live imaging analysis revealed that the T7 transcription track exhibits less mobility and appears to promote colocalization of its flanking regions. In addition, the regions flanking native active genes and transcribed T7 TU tend to (re)localize along the lateral edges of the nucleoid. These experiments suggest that, in *E. coli*, local transcription modulates DNA localization^17,18,24^, while imposing a mechanical constraint on neighboring loci by bringing them closer together^38,39^, and possibly also affecting mobility.

## Discussion

The thinner grain scale made available by resolution improvements, combined with the analysis of native and artificial single transcription unit(s), suggests that transcription shapes bacteria chromosomes by imposing local constraints with multilevel consequences. First, we demonstrate that the large CIDs identified from the long HEG are the tip of a more general phenomena, also visible in the high-resolution Hi-C contact maps of another bacterium (for example, *Vibrio cholerae*, Fig. 4k)^19^. Transcription locally stimulates the formation of bundled domains (TIDs) and promotes contacts between adjacent active genes or operons separated by a few tens of kb (Fig. 4l). Transcription induced by highly expressed artificial T7 promoters also display bundled domains in Hi-C maps and striking arched stripe patterns in 5′ position, which most likely correspond to negative supercoils constrained in-between the promoter and the first quarter of the T7 TU. Furthermore, by combining two divergent T7 TUs, a self-interacting domain appears in the untranscribed region between them. The differences observed between native genes and T7 UT may result from inherent differences between the polymerases, but could also reflect an amplification effect of T7 activity on native processes that are either indistinguishable with current technologies, or overridden by other activities.

We propose that this is the case for the 5′ arched stripe of T7 TU that appears linked to supercoiling. This pattern is manifest at the T7 TSS in absence of neighboring transcription (Fig. 2b), but not so much in the presence of transcription or at the level of active endogenous *E. coli* genes. This suggests that this signal probably reflects dramatic DNA underwounding following strong T7 RNA Pol transcription, which topoisomerase I fails to counteract. In agreement, the divergent or collinear orientation of pairs of T7 promoters can strongly affect the arched stripe patterns, by blurring or alleviating them, respectively. This could explain why this pattern is not observed along native *E. coli* TIDs, since the density, expression level and orientation of these regions may result in similar effects. Therefore, we propose that the constraints imposed by transcription along the fiber balance each other to modulate chromosome organization and dynamics. Note that this arched pattern is therefore different from the ‘stripes’, ‘flames’ or ‘lines’ probably generated through SMC-mediated loop extrusion mechanisms in other species^40–42^. However, transiently these constraints could have multiple consequences for DNA transactions, including transcription, DNA repair and segregation, and contribute as well to the regulation of the extrusion of large DNA loops by bacteria condensins as they travel along the chromosome^7,43,44^.

Sub-kb resolution Hi-C further reveals plaid-like patterns corresponding to contacts between neighboring active endogenous TUs separated by inactive regions (Fig. 1b and Extended Data Fig. 4a), a new feature of bacterial chromosome folding. Furthermore, T7 TUs facing towards *OriC* (that are slightly less strong than TU facing the terminus) also display long-range *trans* contacts with neighboring endogenous active transcription unit upstream the promoter (Extended Data Fig. 3a), suggesting that this pattern is not specific to the *E. coli* polymerase. Since these distant contacts (~20–40 kb) can involve protein-coding genes (membrane and cytoplasmic proteins) and transfer RNA regulated by different transcription factors and different sigma factors (Fig. 1b,d), they may only rely on transcription. Several hypotheses may explain this phenomenon. Firstly, the decreased mobility of transcribed units (Fig. 4j), in association with either relocalization to the nucleoid periphery (Fig. 4c,e,f and ref. ^17^), and/or into cluster of active genes (as suggested in ref. ^24^), could explain such inter-TU contacts (as schematized on Fig. 4l). Secondly, the proximity of transcribing RNA Pols may favor protein–protein interactions as biomolecular condensates^45^. In addition, RNA production may locally reduce effective solvent quality of the cytoplasm and drive local chromosome deformation as proposed by the group of Christine Jacobs-Wagner^18^. Alternatively, or in combination, contacts between adjacent TUs may also be modulated by loop extrusion by the *E. coli* condensin MukB^43^. Loops would extend until they encounter actively transcribed regions that would act as permissive roadblocks or extrusion slowing zones, resulting in enriched contacts between them. Future experiments will be required to assess the contribution(s) of these elements to the folding of transcribed units.

TIDs structural features depend on both transcription level and genomic context, demonstrating that all loci along the chromosome are not subject to transcription-induced mechanical stresses in the same way. For instance, TIDs should form the center of twin-supercoiled domains^4^ recently described using psoralen crosslinking detectable around HEGs (that is, ribosomal operons)^5^ that will span ±25 kb. The relationship between the basic structuring elements that are TIDs and higher-level features of chromosome organization (for example, plectonemic loops^9,10^, macrodomains^46^ and supercoiling domains^5^) is not deciphered in *E. coli*. However, it has been shown that HEG (that results in strong TIDs) represent permissive barriers to cohesin loop extrusions in other species such as *Bacillus subtilis*^43^. A potential influence of TIDs on the *E. coli* SMC MukB could emerge in the future. Also, HEG (for example, ribosomal operons) are frequent in the *oriC* proximal part of the genome, and the resulting TIDs and associated pronounced ‘plaid-like’ patterns may influence *oriC* region folding revealed by Hi-C^13,14^, recombination^46^ and imaging^14^ or supercoiling of this region^5^. This pattern falls within the more general propensity of all adjacent expressed sequences to contact each other more frequently, and thus this behavior would not be specific but only magnified at ribosomal DNA operons.

In eukaryotes, transcription shapes chromosome architecture but the contact patterns differ, with active genes delineating clear boundaries in contact maps for instance in *Saccharomyces* *cerevisiae* (Fig. 4k), as shown by past and recent work^26,47^. In this species, the average gene size is ~1.4 kb, and few genes are larger than 5 kb. As a consequence, it is also possible that the bundle is much shorter and less visible than in bacteria where operons are on average ~3–5 kb in size, and sometimes larger. This pattern appears modulated by SMC complex DNA translocase activity^37,47^. The presence of nucleosomes in eukaryotes and in some archaea is also expected to thicken the contact pattern at short distance, therefore blurring the crisper signal observed in bacteria. Nevertheless, the underlying constraints unveiled in this work imposed by transcription on the DNA sequence stand to be a fundamental aspect of chromosome biology.

## Methods

### Media culture conditions and strains

Strains used in this study are derived from MG1655 and BW25113 *E. coli* strains. They are listed in Extended Data Table 2. All strains were grown in minimal media A (0.26 M KH_2_PO_4_, 0.06 M K_2_HPO_4_, 0.01 M tri sodium citrate, 2 mM MgSO_4_, 0.04 M (NH_4_)_2_SO_4_) supplemented with 0.2% of casamino acids and 0.5% of glucose at 37 °C. BW25113 strains were grown with 0.2% arabinose for 2 h to induce T7 RNA Pol expression under the control of the PBAD promoter. TopA overexpression was also controlled by arabinose for 2 h.

### Drugs and antibiotics

Rifampicin was used for 10 min at a 100 µg ml^−1^ working concentration to inhibit transcription. Novobiocin was used for 10 min at a 50 µg ml^−1^ working concentration to inhibit gyrase. Chloramphenicol was used for 10 min at a 30 µg ml^−1^ working concentration to inhibit translation. When required, ampicillin was used at 100 µg ml^−1^.

### Western blot

Bacteria were resuspended in Laemmli buffer at 2.00 × 10^6^ cells µl^−1^. Protein extracts were ran on 7.5% gel and then transferred to a membrane that was saturated with 10% TBS-T and then labeled with anti-TopA antibodies (mouse antibodies, gift from Yuk-Ching Tse-Dinh) and finally revealed with horseradish peroxidase-coupled anti-mouse antibodies. Revealing was carried out on the femto with the FX fusion device. Membranes were stripped, saturated and labeled with horseradish peroxidase-coupled anti-RpoB antibodies (a loading indicator of protein quantity). Revelation was performed at pico using the FX fusion device. For quantification, the gray level was calculated for each protein, the background was subtracted and the amount of TopA was normalized to the amount of protein (loading control, RpoB).

### Hi-C procedure and sequencing

Cell fixation with 3% formaldehyde (Sigma-Aldrich, cat. no. F8775) was performed as described in ref. ^49^. Quenching of formaldehyde with 300 mM glycine was performed at 4 °C for 20 min. Hi-C experiments were performed as described in ref. ^49^. Samples were sonicated using Covaris (DNA 300 bp).

### ChIP–seq and RNA-seq experiments

Chromatin immunoprecipitation was performed as described^50^. Briefly, overnight cultures were diluted to OD_600nm_ of 0.01, grown until OD_600nm_ of ~0.2–0.25, diluted and crosslinked using formaldehyde (Sigma-Aldrich; final concentration of 1%) for 10 min at 22.5 °C. Formaldehyde was then quenched by adding 2.5 M glycine (final concentration 0.5 M), for 10 min at room temperature (for example, 19–22 °C). Cells were collected by centrifugation at 1,500*g* for 10 min and washed three times in ice-cold 1× phosphate-buffered saline. The pellets can be stored at −80 °C or used straight away. A pellet was then resuspended into 500 μl of 1× TE buffer, supplemented with 5 μl of ready-lyse lysozyme, and incubated with shaking at 37 °C for 30 min. Then 500 μl of 2× ChIP buffer (50 mM HEPES–KOH pH 7.5, 150 mM NaCl, 1 mM ethylenediaminetetraacetic acid (EDTA), 1% Triton X-100, 0.1% sodium deoxycholate, 0.1% sodium dodecyl sulfate and 1× Roche Complete EDTA-free protease inhibitor cocktail) was added, and the sample was transferred to ice. The sample was transferred to a prechilled 1-ml Covaris tube (Covaris), and sonicated using Covaris S220 for 7 min (settings as followed: target size, 200–700; Peak Incident Power 140; Duty Factor 5%; Cycle Per Burst 200). One-hundred microliters of the sample was removed as input and stored at 20 °C. Immunoprecipitation was performed overnight under rotation at 4 using 1/100 T7RNA antibody (Biolabs CB MAB-0296MC) and antiflag (Sigma F1804 and F3165). Immunoprecipitated samples were incubated with Protein G Dynabeads (Invitrogen) with rotation for 2 min at room temperature. The tube was washed three times with 1× phosphate-buffered saline with 0.02% Tween-20 using the Dynamag magnet setup. The beads were resuspended in 200 μl TE buffer with 1% sodium dodecyl sulfate and 1 μl RNAseA (10 mg ml^−1^) and 1 μl proteinase K (20 mg ml^−1^). Samples were incubated at 65 °C for 10 h to reverse the formaldehyde crosslinking. The beads were removed using the Dynamag magnet and DNA of the supernatant purified using Qiagen Minelute polymerase chain reaction (PCR) purification kit using two elution steps. DNA was eluted into a 50 μl TE buffer and stored at −20 °C until further processing.

### RNA-seq

Total RNA was extracted from *E. coli* using the Nucleospin RNA Extraction Kit (Macherey-Nagel) according to the manufacturers’ instructions. DNAse was depleted using an additional DNase treatment with Turbo DNase (Thermo Fisher). The DNAse was inactivated and RNA was purified by a phenol–chloroform extraction (pH 4.5, Amresco) and ethanol precipitation. The RNA was then resuspended in diethyl pyrocarbonate-treated water. Ribosomal RNA depletion was done using Ribo-Zero magnetic beads according to the manufacturer’s protocol (Illumina). Complementary DNA library preparation was performed following standard protocols. Briefly, RNA was fragmented using the NEBnext mRNA first and second strand synthesis kits (NEB). One to three biological replicates were generated for each condition, and on average ~10 million reads were generated per sample.

### DNA libraries preparation

For Hi-C, RNA-seq and ChIP–seq libraries, preparation of the samples for paired-end sequencing was performed using Invitrogen Colibri PS DNA Library Prep Kit for Illumina according to the manufacturer’s instructions. The detailed protocol is available in ref. ^49^. All libraries used or generated during the course of this study are listed in Extended Data Table 3.

### Gradient preparation of *E. coli* polysomes

To preserve the polysomes, cultures of *E. coli* are incubated with 100 µg ml^−1^ of chloramphenicol before centrifugation. Fresh cell paste (0.7 g) was homogenized in the buffer (150 mM NH_4_Cl, 10 mM MgCl_2_, 2 mM Tris–Cl pH 7.5, 10 μM phenylmethylsulfonyl fluoride and 0.2 µg ml^−1^ chloramphenicol, Complete EDTA-free, RNAsine) at a 1:2 (w:v) ratio and set aside for 20 min at 4 °C. Disrupt cells using FastPrep sample preparation system and lysing matrix B tubes (2 ml) containing 0.1 mm silica beads. Add sodium deoxycholate (1% final), DNase I to a final concentration of 2 µg ml^−1^ (20 U ml^−1^) and let 30 min on ice, then clear the lysate of cell debris by centrifugation at 4 °C using benchtop centrifuge for 20 min and a second centrifuge for 5 min. Divide the supernatant equally, and treat one part by adding EDTA (70 mM) and incubate on ice for 30 min. Layer the fractions (600 µl) on top of 10 ml sucrose gradient (10–40%) and centrifuge for 2.5 h at 4 °C in SW41Ti rotor at 35,000 rpm (151,000*g*). Gradients are next fractionated by collecting 500-µl fractions. To analyze RNA, 170 µl of each fractions is mixed to 400 µl of RNAse-free water and 570 µl of phenol, vortexed and centrifuged to extract RNA from proteins, then aqueous supernatant is precipitated with CH_3_COONa, glycogen and isopropanol. Collected RNA present in each fraction is next analyzed in agarose gel.

### Processing of reads and Hi-C data analysis

Reads were aligned with bowtie2 v2.4.4 and Hi-C contact maps were generated using hicstuff v3.0.3 (ref. ^51^) with default parameters and using HpaII enzyme to digest. Contacts were filtered as described in ref. ^22^, and PCR duplicates (defined as paired reads mapping at exactly the same position) were discarded. Matrices were binned at 0.5, 1, 2 or 5 kb. Balanced normalizations were performed using ICE algorithm^52^. Reads with ambiguous mapping were removed such as reads mapping on the rDNA operons, resulting in missing values into the Hi-C contact map (white lines). Contact maps are stored in cool file format using cooler (v0.8.11)^53^. For all comparative analyses, matrices were downsampled to the same number of contacts. Comparison between matrices was done using log_2_ ratio and serpentine v0.1.3 (ref. ^48^) for flexible binning. Serpentine was used with 5-kb binned matrices, with 25 iterations and a threshold of 100. The Hi-C signal was computed as the contacts between adjacent 5 kb bins as described in Lioy et al.^13^. To compare this signal with other genomics tracks, we binned it at the desired resolution and *z*-transformed it.

### Border detection

To detect the borders we first used the directional method as described in ref. ^13^. The directional index is a statistical parameter that quantifies the degree of upstream or downstream contact bias for a genomic region^54^. For each bin, we extracted the vector of contacts from the correlation matrix between that bin and bins up to a window size in both left and right directions. To assess if the strength of interactions is stronger with one direction relative to the other we used a paired *t*-test between the two vectors. A *P* value of 0.05 was used as a threshold to assess a statistical significant difference. The directional preferences for the bin along the chromosome are represented as a bar plot with positive and negative *t* values shown as red and green bars, respectively. We trimmed the bars of the bins with *t* values below −2 or above 2 (corresponding to a *P* value of 0.05). At the borders identified in the contact matrices, the directional index changes from negative to positive *t* values. The implementation of the code is available at ref. ^55^, v1.0.1, and it is based on the one used for Lioy et al.^13^. The DI method depends on the binning resolution and on the window size. At small window size, it misses the larger domains visible at larger scale, and at large window size it finds only the larger domains. Moreover, the resolution impacts on the performance of the DI: at low resolution it cannot find the smallest domains that are merged in few bins, and at high resolutions it starts to be noisy as the resolution directly impacts the width of the vectors used to compute the DI. In our study, we decided to use an insulation score method to improve the borders detection at higher resolution. For our analysis, we developed a python implementation^55^ of the HiCDB algorithm^25^. This method allows multiple window sizes, which reduces the dependence between the window size and the size of the detected domains. Furthermore, it does not depend on the resolution of the matrix, which allows for efficient detection of boundaries even at high resolution. We used the 1 kb resolution contact map with 10, 15, 20, 25 and 30 kb windows (Extended Data Table 1).

### Pileup analysis

The Hi-C contacts were built and normalized as explained before at a resolution of 500 bp. For each gene we extract a 100 kb matrix centered on the start codon of the gene. For reverse genes, we flip the matrix to have the centered genes pointing always in the same direction. The pileup plot is the average of all the extracted windows, without taking into account the white lines (that is, bins with less than the median minus three times the median absolute deviation are considered as white lines). To select active genes, we select a fraction of the most transcribed genes (values in reads per kilobase per million) as the active genes. For the transcription units analysis, to center our windows on the first transcribed genes, we selected active genes only if there are no other active genes in the 3 kb upstream of the start codon of the gene. To compare the pileups of the first transcribed genes with the noncoding or nontranscribed regions, we calculated the ratio between the pileup of the first transcribed genes and the pileup of random windows taken from the same region (center on a random position within 100 kb around the gene). We chose to use random regions instead of the pileup of noncoding genes or the expected matrix (matrix corresponding to the contacts of the genomic distance law) to avoid having a bias of the region where we extract the active genes.

### Detection of contact bundles (that is, TIDs) along the main diagonal

To detect contact bundles on the main diagonal, we used a convolution kernel on the balanced matrix. The method is implemented in ref. ^55^. We used a computer vision approach similar to the program Chromosight^56^, which uses a convolution kernel describing a given pattern as a template to detect the local similarity with it. Here we aim at detecting the bundles on the main diagonal of the matrix. To detect them, we build a gaussian kernel of size *n* as follows (*n* = 5 in our study):

$${M}_{i,j}=\frac{1}{\surd 2}{e}^{{-\frac{1}{2}\left[\frac{{\rm{|}}i-j{\rm{|}}+{\rm{|}}n-1-i-j{\rm{|}}}{2(n-1)}\right]}^{2}}$$

By computing the convolution product between each local image centered on each bin of the main diagonal and the kernel, we obtain a convolution score. The higher the score is, the closer the local image is to the kernel and the more likely it is to be a bundle. To remove the effect of local regions, we remove the second envelope of the signal as it’s described in the HiCDB insulation score algorithm^25^. Finally, the borders of the bundles are detected by taking each peak of the local convolution score superior to the median of the local convolution score. The bundle region is then extended until the value gets inferior to one-third of the peak.

### RNA-seq processing

Processing is done using tinymapper v0.10.0 (ref. ^57^) with default RNA parameters. The reads are mapped with bowtie2 v2.4.4, PCR duplicates are filtered using samtools v1.14 and count per million (CPM) is made with bamCoverage v3.5.1. We used only the unstranded signal, and binned it depending on the displaying resolution. For the comparisons with other signals, a *z*-transformation is done.

### ChIP–seq processing

Processing of the ChIP–seq of T7 RNA Pol and GapR is done using tinymapper v0.10.0 (ref. ^57^) with default ChIP–seq parameters without input. The reads are mapped with bowtie2 v2.4.5, PCR duplicates are filtered using samtools v1.15 and CPM is made with bamCoverage function from deeptools (v2.29.1). For the GapR-seq, we do a gaussian blur of the signal with the gaussian_filter1d function from scipy v1.7.3 with ‘wrap’ mode and sigma value of 2,500, as described in Guo et al.^35^. The data are then binned at the displaying resolution and *z*-transformed to compare it to other signals.

### Imaging and analysis

Cells were grown similarly to Hi-C samples (above). One hour after arabinose induction of T7 RNA Pol, 2 ml of cells were pelleted and resuspended in 50 µl of fresh medium. Three drops of 2 µl are deposited on a freshly made agarose pad (1× supplemented medium A, 1% agarose) incubated 30 min in the microscope incubation chamber at 30 °C and imaged. For foci mobility analysis, imaging was performed on a Nikon Eclipse Ti inverted microscope equipped with a Spinning-Disk CSU-X1 (Yokogawa), an EM-CCD Evolve 512*512, magnification lens 1.2, pixel size: 13.3 µm × 13.3 µm camera at 600-fold magnification. Focal plane was maintained during acquisition using Nikon Hardware autocus. Illumination and acquisition was controlled by Metamorph. Imaging was perfomed for 120 s every second with a 100 ms acquisition time. Time series images were registered using Stackreg^58^ and analyzed with the MOSAIC suite^59^ as FIJI plugins. Median MSD (*α*) distribution was analyzed and plotted with Graphpad-PRISM. An average of 1,000 trajectories were analyzed for each replicate. For interfocal distances and nucleoid organization measurements, cells were observed live on agarose pad on a thermo-controlled stage with an epifluorescence-LED system mounted on a Zeiss inverted confocal microscope and a C-MOS Hamamatsu 2,048 × 2,048/pixel size: 6.45 × 6.45 µm camera at 630-fold magnification. The position of foci in the cell in each condition was analyzed with the ObjectJ plugins of ImageJ^60^. Two-color localization was performed with *cr*::*parS*^*P1*^ and *yajQ*::*parS*^*pMT1*^ tags^61^. An average of 1,200 cells were analyzed per strain and condition. Distributions were analyzed and plotted with MATLAB. Confidence intervals on the plot were made using a bootstrap of sampling of the original values. The *P* values were computed using a Kolmogorov–Smirnov test.

### Modeling approach

We devised a simple model to reproduce the contact maps obtained experimentally under a few hypotheses. We start our approach by computing the contact probability decline with increasing genomic distance from experimental data p(*s*). We then make the hypothesis that two different types of contacts are found in the experiments: contacts mediated by polymerases and contacts mediated by other proteins. We assume that the proportion of contacts mediated by polymerases at bin *i* is *C*_*i*_, where *C*_*i*_ is the normalized experimental Pol-ChiP signal. To normalize the signal, we define its maximum value as *ε*, which is between 0 and 1. The proportion of contacts mediated by other proteins at bin *i* is then simply 1 − *C*_*i*_. We then compute the contact probability between any couple of bins *i* and *j* using two different models:

- In model 1, there is a preferential interaction between polymerases so that the contact frequency is proportional to: p(*s*_*i*,*j*_) × (*C*_*i*_*C*_*j*_ + (1 − *C*_*i*_)(1 − *C*_*j*_)
- In model 2, there is a preferential interaction only between consecutive polymerases. The idea behind this model is that polymerases also act as contact insulators. The contact frequency is then modified from model 1: p(*s*_*i*,*j*_) × (*m* × *C*_*i*_*C*_*j*_ + (1 − *C*_*i*_)(1 − *C*_*j*_)) with ${m}={\sum }_{i+1}^{j-1}{Cn}.$ *m* represents the insulation factor, which is proportional to the total amount of polymerase that is found between bins *i* and *j*.

After all contact probabilities have been computed for each model, the contact matrix is normalized so that the sum of each line and each column is equal to 1 so that it corresponds to contact probabilities. The Spearman rank correlation is then computed between the experimental map, and the model map is then computed to find the best value for epsilon and to compare the relevance of each of the two models.

### Reporting summary

Further information on research design is available in the Nature Portfolio Reporting Summary linked to this article.

## Online content

Any methods, additional references, Nature Portfolio reporting summaries, source data, extended data, supplementary information, acknowledgements, peer review information; details of author contributions and competing interests; and statements of data and code availability are available at 10.1038/s41594-023-01178-2.

### Supplementary information

Reporting Summary

### Source data

Source Data Extended Fig. 4 Extended Data Fig. 4b: imaging of bacteria overexpressing Topol or not.

Source Data Fig. 4 Normalized longitudinal and lateral positions of the foci in cells containing 1 *parS*^*P1*^ and 1 *parS*^*pMT1*^ foci. Proportion of cell with colocalized *parS*^*P1*^ and *parS*^*pMT1*^ foci. Median MSD slope of 1,000 foci for each replicate. MSD *α* slope of more than 1,000 lacI–YFP trajectories for each replicate.

Source Data Extended Fig. 4 Unprocessed western blot gels.

## Figures and Tables

**Extended Data Table 1:**
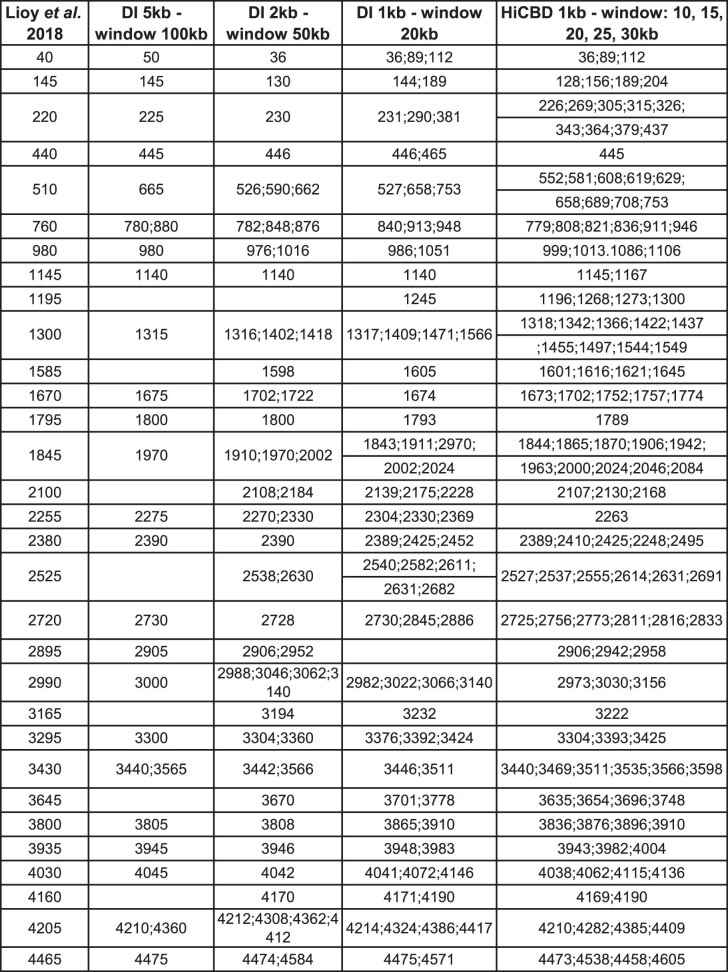
Positions of the detected borders

Borders from the different methods and Lioy et al. 2018^8^.

**Extended Data Table 2:**
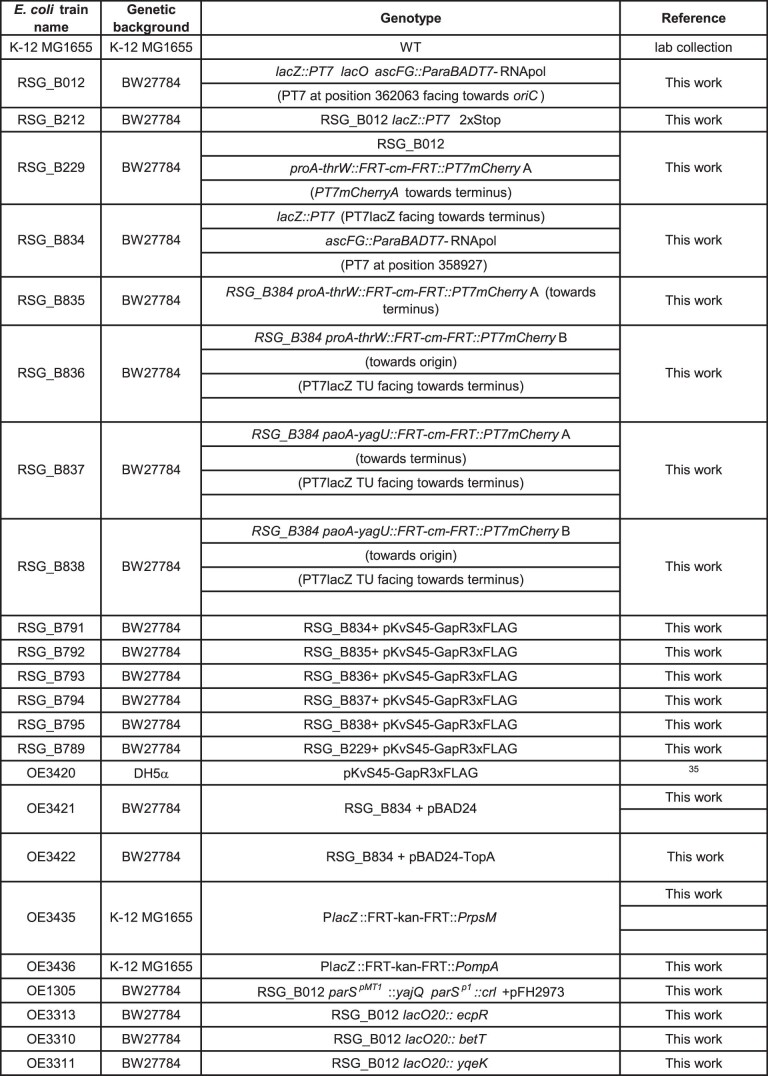
Strains used in the study

Description of the different genotype used.

**Extended Data Table 3:**
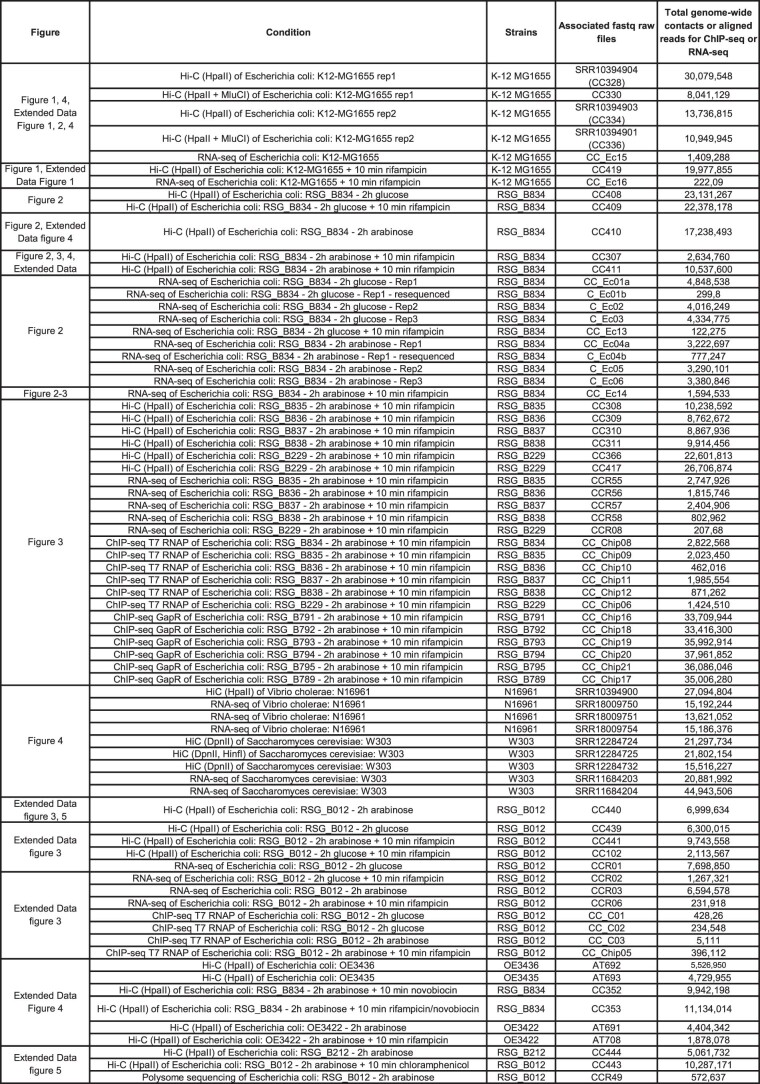
Libraries used in this study

Data are available at PRJNA844206.

## Data Availability

The accession number for the sequencing reads reported in this study is PRJNA844206. The reference genome for *E. coli* K12 MG1655 strain, GCF_000005845.2, is provided at https://www.ncbi.nlm.nih.gov/assembly/GCF_000005845.2, and for *V. cholerae* O1 El Tor N16961, GCF_003063785.1, at https://www.ncbi.nlm.nih.gov/assembly/GCF_003063785.1. For *S. cerevisiae*, the reference genome of the W303 strain used is available at https://www.ncbi.nlm.nih.gov/assembly/GCA8002163515.1. Microscopy data are available at Mendeley Data, V1, at https://data.mendeley.com/datasets/fzrmgjfyg7/1. Source data are provided with this paper. Strains of this study are available from the corresponding authors.

## Code availability

The custom-made code of the analysis is available at https://github.com/koszullab/transcription_T7_analysis. Open-access versions of the programs and pipeline used are available online.
