# Supplementary material for: Transcription-induced domains form the elementary constraining building blocks of bacterial chromosomes
Source: Nat Struct Mol Biol. 2024 Jan 4;31(3):489–97. doi: 10.1038/s41594-023-01178-2 (PMC10948358; doi:10.1038/s41594-023-01178-2)
Supplement: Supplementary file 1 — Reporting Summary [file 41594_2023_1178_MOESM1_ESM.pdf]

nature portfolio

Corresponding author(s): Olivier Espeli, Romain Koszul

Last updated by author(s): Oct 30, 2023

Reporting Summary

Nature Portfolio wishes to improve the reproducibility of the work that we publish. This form provides structure for consistency and transparency in reporting. For further information on Nature Portfolio policies, see our Editorial Policies and the Editorial Policy Checklist.

Statistics

For all statistical analyses, confirm that the following items are present in the figure legend, table legend, main text, or Methods section.

n/a	Confirmed	
☐	☒	The exact sample size (*n*) for each experimental group/condition, given as a discrete number and unit of measurement
☐	☒	A statement on whether measurements were taken from distinct samples or whether the same sample was measured repeatedly
☐	☒	The statistical test(s) used AND whether they are one- or two-sided<br>*Only common tests should be described solely by name; describe more complex techniques in the Methods section.*
☒	☐	A description of all covariates tested
☐	☒	A description of any assumptions or corrections, such as tests of normality and adjustment for multiple comparisons
☐	☒	A full description of the statistical parameters including central tendency (e.g. means) or other basic estimates (e.g. regression coefficient) AND variation (e.g. standard deviation) or associated estimates of uncertainty (e.g. confidence intervals)
☐	☒	For null hypothesis testing, the test statistic (e.g. *F*, *t*, *r*) with confidence intervals, effect sizes, degrees of freedom and *P* value noted<br>*Give P values as exact values whenever suitable.*
☒	☐	For Bayesian analysis, information on the choice of priors and Markov chain Monte Carlo settings
☒	☐	For hierarchical and complex designs, identification of the appropriate level for tests and full reporting of outcomes
☐	☒	Estimates of effect sizes (e.g. Cohen's *d*, Pearson's *r*), indicating how they were calculated

Our web collection on statistics for biologists contains articles on many of the points above.

Software and code

Policy information about availability of computer code

Data collection: No software have been used.

Data analysis: All the scripts used in this study are available in the following github repository: https://github.com/koszullab/T7_promoter_analysis
The programs involved in this study. The following programs are the one cutsom made or open sources programs from Koszul laboratory:
hicstuff v3.1.4 (available at https://github.com/koszullab/hicstuff)
bacchus v1.0.1 (available at https://github.com/ABignaud/bacchus)
TinyMapper v0.10.0 (available at https://github.com/js2264/tinyMapper)
serpentine v0.1.3 (https://github.com/koszullab/serpentine/tree/master)

As well as programs developed and published by others:
bowtie2 v2.4.5 (available at https://github.com/BenLangmead/bowtie2)
samtools v1.15 (available at http://www.htslib.org/)
deepTools v2.29.1 (available at https://github.com/deeptools/deepTools)
cooler v0.8.11 (available at https://github.com/open2c/cooler)
scipy v1.7.3 (available at https://scipy.org/)

For manuscripts utilizing custom algorithms or software that are central to the research but not yet described in published literature, software must be made available to editors and reviewers. We strongly encourage code deposition in a community repository (e.g. GitHub). See the Nature Portfolio guidelines for submitting code & software for further information.

Data

Policy information about availability of data

All manuscripts must include a data availability statement. This statement should provide the following information, where applicable:

- Accession codes, unique identifiers, or web links for publicly available datasets
- A description of any restrictions on data availability
- For clinical datasets or third party data, please ensure that the statement adheres to our policy

Sequencing datasets for all figures have been deposited in SRA under the accession code of PRJNA844206

Reference genomes:
- E. coli K12 MG1655: https://www.ncbi.nlm.nih.gov/nuccore/NC_000913.3/
- V. cholerae O1 El Tor N16961: https://www.ncbi.nlm.nih.gov/data-hub/genome/GCF_000006745.1/
- S. cerevisiae W303: https://www.ncbi.nlm.nih.gov/assembly/GCA8002163515.1/

Microscopy data are available at Mendeley Data, V1, doi: 10.17632/fzrmgjfyg7.

Source data are provided with this paper.
Strains of this study are available from the corresponding authors.

Human research participants

Policy information about studies involving human research participants and Sex and Gender in Research.

Reporting on sex and gender	NA
Population characteristics	NA
Recruitment	NA
Ethics oversight	NA

Note that full information on the approval of the study protocol must also be provided in the manuscript.

Field-specific reporting

Please select the one below that is the best fit for your research. If you are not sure, read the appropriate sections before making your selection.

☒ Life sciences ☐ Behavioural & social sciences ☐ Ecological, evolutionary & environmental sciences

For a reference copy of the document with all sections, see nature.com/documents/nr-reporting-summary-flat.pdf

Life sciences study design

All studies must disclose on these points even when the disclosure is negative.

Sample size	No samples size calculation were performed: experiments are bulk and each measurement reflects millions of cells. The experimental design follows the standard guidelines and procedures in the field (i.e. similar samples sizes, incubation time, etc.). The sample size (n) of independent experiments is provided in the methods of the manuscript. Sample sizes were chosen to support meaningful conclusions. See following publications for similar guidelines: Lioy et al., Cell, 2018 ; Cockram et al., Mol Cell, 2021.
Data exclusions	No data were excluded from the analysis.
Replication	4 biological HiC replicates have been used for the WT HiC on E. coli. For the RNA-seq experiments with one T7 prometer in the forward strand 3 replicates have been done for the library without rifampicine and only one for the library with rifampicine as we just want to control that transcription is shot down. For other genomics libraries we did only one replicate.<br>the microscopy analysis of the positionning of the two parS sites have been made based on between 796 and 1823 cells in each strain and each condition. Between 7 to 9 biological replicates of each strain and conditions (different cultures at different time) have been pooled.<br>To compute the trajectories of the locus, between 3 and 6 replicates have been made for each strain and contitions to have at the end the trajectories of approximately 1000 cells for each.<br>Different numbers of replicates have been made to have the similar number of cells once it's pooled.<br>All attempts at replication were successful.
Randomization	No randomization of samples was relevant nor performed: randomization is not relevant because we did not use experiment groups in our

Randomization	study.
Blinding	The experiments were not blinded: we did not have experimental groups to compare. Investigators were blinded during data analysis.

Reporting for specific materials, systems and methods

We require information from authors about some types of materials, experimental systems and methods used in many studies. Here, indicate whether each material, system or method listed is relevant to your study. If you are not sure if a list item applies to your research, read the appropriate section before selecting a response.

Materials & experimental systems

n/a	Involved in the study	
☐	☒	Antibodies
☒	☐	Eukaryotic cell lines
☒	☐	Palaeontology and archaeology
☒	☐	Animals and other organisms
☒	☐	Clinical data
☒	☐	Dual use research of concern

Methods

n/a	Involved in the study	
☐	☒	ChIP-seq
☒	☐	Flow cytometry
☒	☐	MRI-based neuroimaging

Antibodies

Antibodies used

ChIP-seq antibodies:
- T7 RNA pol antibody (Biolabs CB MAB-0296MC) for the T7 RNA pol ChIP-seq diluted 1/1000.
- Mouse antiflag (Sigma F1804 clone M2 and F3165) for the GapR ChIP-seq
- Mouse monoclonal antibody against E.coli TopA antibody (gift from Tse-dinh) used as described in Zhou, et al 2017 NAR for the western-blot diluted 1/1000
- HRP:
Primary antibody : anti-E. coli RNA Polymerase β Antibody (Biolegend 663907) for the western blot diluted 1/2000
Secondary antibody : HRP anti-mouse antibody for the western-blot diluted 1/10000

Validation

- Mouse anti-FLAG (Sigma Aldrich, F1804 clone M2) is validated on Sigma Aldrich website.
http://www.sigmaaldrich.com/FR/fr/product/sigma/f1804
GapR ChIP-seq profiles yield expected values as the ones previously published (see Freddolino et al., PLOS Biol., 2021).
- T7 RNA pol antibody was test by Western blot.
https://www.creativebiolabs.net/Anti-T7-RNA-Polymerase-Antibody-78687.htm?gclid=CjwKCAjwsfuYBhAZEiwA5a6CDIZnSVjE0dkEZ6ZQFHIih3oH1D_oTvWoOWe1uvWJ8H5QfHvC7VkYeBoC9oIQAvD_BwE
- The anti-TopA monoclonal antibodies were generated in the 1990's by Rolf Menzel at Bristol-Myers Squibb for a project on topA transcription. TopA antibodies were used previously in the study: Direct interaction between Escherichia coli RNA polymerase and the zinc ribbon domains of DNA topoisomerase I.
Cheng B, Zhu CX, Ji C, Ahumada A, Tse-Dinh YC. J Biol Chem. 2003 Aug 15;278(33):30705-10. doi: 10.1074/jbc.M303403200. Epub 2003 Jun 4. PMID: 12788950
- HRP : (https://urldefense.com/v3/__https://www.biolegend.com/en-gb/search-results/direct-blot-hrp-anti-e-coli-rna-polymerase-beta-antibody-13499?GroupID=GROUP26__;!!JFdNOqOXpB6UZW0!qTAVjUvvrh3s1B3zQHMllHLTERap_lDp2ggew3F0GuWiCoXzaBAJl4cJZ_wb8dS-xUnYyIkGdESvVg-c1rm_dBvb-q7jKs8mENCv96RO$)

ChIP-seq

Data deposition

☒ Confirm that both raw and final processed data have been deposited in a public database such as GEO.

☒ Confirm that you have deposited or provided access to graph files (e.g. BED files) for the called peaks.

Data access links<br>*May remain private before publication.*	https://www.ncbi.nlm.nih.gov/bioproject/PRJNA844206<br>https://www.ncbi.nlm.nih.gov/geo/query/qcc.cgi?acc=GSE213028
Files in database submission	Filenames included in the ChIP-seq and HiC database submission are provided in the supplemnatry table 3: "Libraries used in this study available at PRJNA844206."
Genome browser session<br>(e.g. UCSC)	No longer applicable.

Methodology

Replicates

The coherence between the different constructions carrying similar features were used as replicates. GapR ChIP-seq profiles yield expected values as the ones previously published (see Freddolino et al., PLOS Biol., 2021).

Sequencing depth	Numbers of reads uniquely mapped and paired on E. coli are reported in the supplementary table 3 (Libraries used in this study available at PRJNA844206.) provided in the manuscript.<br>CC_ChIP08; 5,062,356 reads<br>CC_ChIP09; 5,014,893 reads<br>CC_ChIP10; 5,850,694 reads<br>CC_ChIP11; 5,134,958 reads<br>CC_ChIP12; 5,260,568 reads<br>CC_ChIP06; 3,518,621 reads<br>CC_ChIP16; 21,701,615 reads<br>CC_ChIP18; 21,541,473 reads<br>CC_ChIP19; 23,914,039 reads<br>CC_ChIP20; 25,858,578 reads<br>CC_ChIP21; 24,347,473 reads<br>CC_ChIP17; 22,673,532 reads<br>CC_C01; 24,563,583 reads<br>CC_C03; 5,282,265 reads ; reads aligned<br>CC_ChIP05; 7,200,704 reads ; reads aligned
Antibodies	ChIP-seq antibodies:<br>- T7 RNA pol antibody (Biolabs CB MAB-0296MC) for the T7 RNA pol ChIP-seq<br>- antiflag (Sigma F1804 and F3165) for the GapR ChIP-seq<br>The validation has been described upper in the antibodies section.
Peak calling parameters	No peak calling have been performed: GapR and T7 RNApol yield broad signals and we were not interested on the peak detection.
Data quality	Reads quality was assessed using FastQC. T7 RNA-pol ChIP-seq were concordant with the transcriptomic profiles. GapR ChIP-seq profiles yield expected values as the ones previously published (see Freddolino et al., PLOS Biol., 2021).
Software	Bowtie2 was used for the alignments on the E. coli genome, allowing the generation of an alignment of IP and WCE that exclusevely mapped on the genome. The obtained SAM files were converted into a BAM files, sorted, filtered and indexed using samtools. ChIP-seq profiles were then normalised by the number of million sequences and converted into BigWig using bamCoverage.
