# Supplementary material for: Transcription-induced domains form the elementary constraining building blocks of bacterial chromosomes
Source: Nat Struct Mol Biol. 2024 Jan 4;31(3):489–97. doi: 10.1038/s41594-023-01178-2 (PMC10948358; doi:10.1038/s41594-023-01178-2)

Extended Figure 4b

Well	1	2	3	4	5	6	7	8	9	10	11	12
Condition	RSG_B834 pBAD	RSG_B834 pBAD-TopA		RSG_B834 pBAD-TopA		Marker	RSG_B834 pBAD	RSG_B834 pBAD-TopA				RSG_B834 pBAD-TopA
Dilution	1	5		1			1	4	5	6		1
Deposited volume	15uL	10uL		15uL		4uL	15uL	10uL	10uL	10uL		10uL

RpoB

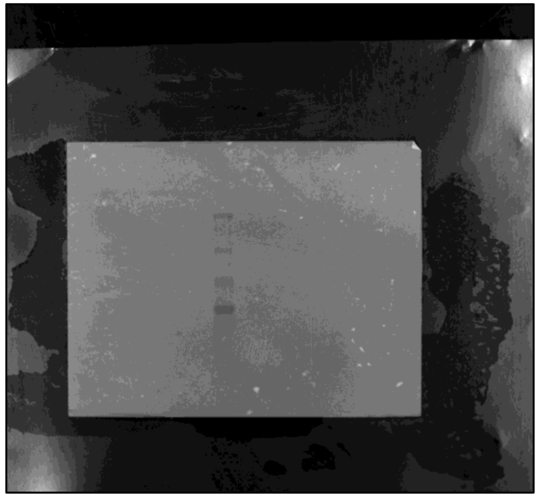

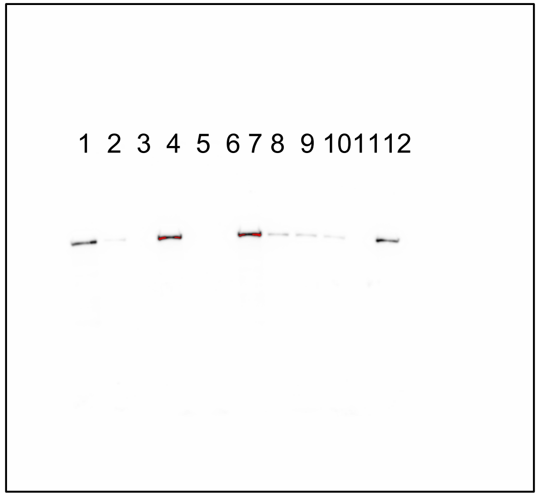

TopA

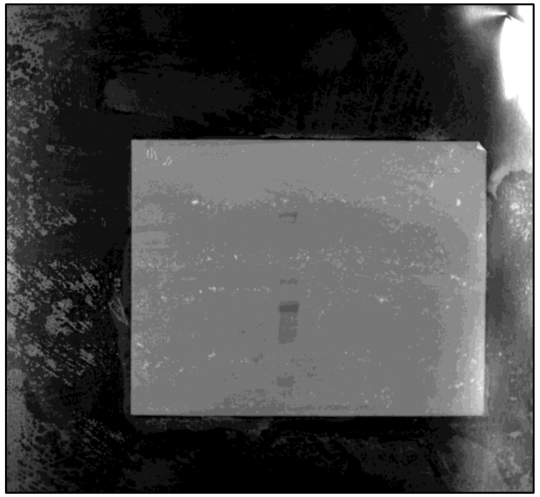

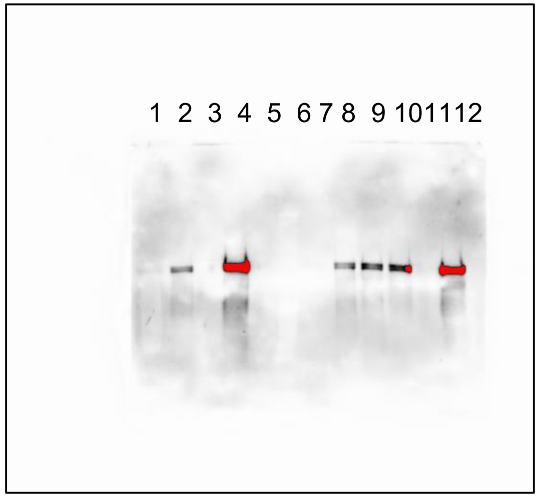

Supplement: Supplementary file 4 — Unprocessed western blot gels. [file 41594_2023_1178_MOESM4_ESM.pdf]
